# Is the use of videotape recording superior to verbal feedback alone in the teaching of clinical skills?

**DOI:** 10.1186/1471-2458-9-474

**Published:** 2009-12-19

**Authors:** Nilgun Ozcakar, Vildan Mevsim, Dilek Guldal, Tolga Gunvar, Ediz Yildirim, Zafer Sisli, Ilgi Semin

**Affiliations:** 1 Dokuz Eylul University Medical Faculty Department of Family Medicine, Izmir, Turkey

## Abstract

**Background:**

In recent times, medical schools have committed to developing good communication and history taking skills in students. However, there remains an unresolved question as to which constitutes the best educational method. Our study aims to investigate whether the use of videotape recording is superior to verbal feedback alone in the teaching of clinical skills and the role of student self-assessment on history taking and communication skills.

**Methods:**

A randomized controlled trial was designed. The study was conducted with 52 of the Dokuz Eylul University Faculty of Medicine second year students. All students' performances of communication and history taking skills were assessed twice. Between these assessments, the study group had received both verbal and visual feedback by watching their video recordings on patient interview; the control group received only verbal feedback from the teacher.

**Results:**

Although the self-assessment of the students did not change significantly, assessors' ratings increased significantly for videotaped interviews at the second time.

**Conclusions:**

Feedback based on videotaped interviews is superior to the feedback given solely based on the observation of assessors.

## Background

As the modern medical perspective evolved from a biomedical paradigm towards a more psychosocial perspective, interviewing the patient and taking their medical history has gained greater importance and significance.

In the 21st century, medical schools aspire to educate physicians who will focus on the patient rather than the disease. Embracing this principle, medical schools are committed to teach all components of good communication and good clinical reasoning skills through the process of interviewing the patient. At the present, the core curriculum of many medical schools contains these subjects within its clinical skills program. These educational programs use a wide range of educational methods including lectures, portfolios, small group practices and role playing in the teaching of clinical skills.

Another important issue in this field is the assessment of the education [1-4]. Simulated patients play a vital role in the doctor patient interview training. This educational method is principally based on observing students during interviews with simulated patients and giving them feedback afterwards either by assessors, simulated patients or peers. Observation may be done by using two-way mirrored rooms, videotape recording or just by direct vision by assessors in the room during the process [5-9]. There are studies that have showed that using videotaped consultation training by way of videotaping real consultations with subsequent feedback, was considered in general as an acceptable, useful, inspiring, powerful and effective teaching tool which could be useful and effective in improving clinical skills [10-12]. Our study aims to investigate:

1) Does feedback involving reviewing videotaped performance on a patient encounter as well as verbal feedback from an assessor, improve subsequent performance on history taking and communication skills more than verbal feedback alone from the assessor?, and 2) What is the role of student self-assessment in subsequent student performance on history taking and communication skills?

## Methods

### Participants

Among the 144 second year students, 52 students who did not take place in the interview practice were selected via a random numbers table and enrolled in the study. 27 of them were placed into the study group, while 25 took part in the control group. Students were informed of the purpose of the study and gave their consent to participate in the study. Ethics committee of Dokuz Eylul University Faculty of Medicine (DEUFM) has given the approval for the study.

### Design

A randomized controlled trial was designed to compare the effects of different feedback methods on the performance of students.

The study was conducted with DEUFM second year students, in the first month of their academic year. Since 2001 the clinical skills program, which includes interviewing the patient, history taking, and performing physical examinations as well as recording skills, has been spread out over the first three years of medical school. The main goal of the clinical skills program is to successfully attain these skills in the preclinical period and to facilitate early contact with the patient. The curriculum consists of the basic perspectives and characteristics of interview and history taking in the first year, learning the skills based on systems in the second year and the synthesis of all these skills in the third year.

### Instruments

The checklist which was used in the assessment was composed of two parts. The first part included 10 variables which evaluated communication skills, and the second part consisted of twelve variables which measured the components of the medical history in the form of a likert-like scale (Additional file 1). While evaluating communication skills and taking history, if the students omitted an item, the result was marked as "unsatisfactory", if the student had questioned only one subheading of each item, the result was marked as "borderline", while if the student had questioned more than one subheading of each item then it was marked as "satisfactory". For example, in "Facilitating skills" the seventh item of the communication scale contains various subheadings such as "Does he/she make eye contact? Is his/her speech clear?" etc. If the student did not use any of the facilitating skills noted, then the result was marked as "unsatisfactory." If he/she only made eye contact, but neglected the other steps, the result was marked as "borderline" and if he/she made eye contact and performed any other of the subheading the result was marked as "satisfactory". The reliability of the communication skills scale was acceptable in medium level (Cronbach's alpha = 0.77). The history taking scale was used in our education program which based on universally used and accepted steps of history taking.

The scenarios of the cases used in our study both related with pain. The first one was headache due to migraine and the second one was low back pain due to herniated disc.

### Procedures

During the first interviews for both groups, assessors observed students and assessed them via a checklist. In the study group, however, in addition to this, the interviews were recorded on video tape. Finally, after the interviews, both student groups were asked to assess themselves using the same checklist. In the control group, the assessors gave only verbal feedback to the subjects. In the study group, on the other hand, feedback was given verbally but after the trainer and student watched the video recording together. After a period of 15 days, the students of both groups interviewed the patients again, however, this time the study group was not video recorded. Students were assessed again by the assessors, and assessed themselves using the same checklist. All subjects attended the same lectures and practices during this two week period.

All students enrolled in the study interviewed simulated patients in two-way mirrored rooms twice. Four family medicine department staff, permanently assigned assessors of clinical skills, made the observations. Interrater reliability was found as Kappa>0.90.

### Data Analysis

22 variables were divided into several subscales. "Communication score" ranged between 0 - 12 and entailed greeting the patient, comforting and determining the level of communication, facilitating skills and using communication skills. "Ability of taking medical history score" ranged between 0 - 18 and included beginning with open ended questions and continuing with closed ended ones, constructing the history in sequence, guiding the patient and summarizing history episodes, determining the process and ending in an appropriate manner. "History of present illness score" ranged between 0 - 15 and included the variables of primary, secondary and tertiary story, the patient's perspective and the things that have been done about the illness. "Other history components score" ranged between 0 - 15 and involved the past medical history, present health condition, family history, personal and social history and reviewing of systems. "Total history score" was the summation of the above history scores plus the main complaint and the identification data. Total history score ranged between 0 - 33.

"Total score" was the sum arrived at by adding total history and total communication scores and ranged between 0 - 66.

The data was analyzed by SPSS 11.0 for windows Statistical Program. Descriptive statistics, and independent and dependent sampling t test was used for group comparisons. Statistical significance was tested at the level of p < 0.05.

## Results

27 of the 52 students enrolled in the study were included in the study group, whereas 25 students comprised the control group. There were 18 male and 9 female students and 14 male and 11 female students in the study and the control groups respectively. There were no significant differences in the distribution of gender between the two groups (Chi^2 ^= 0.624, p = 0.43 > 0.05).

The mean ages of students were 20.80 for the study group and 20.88 for the control group with no significant differences between the groups (p = 0.95 > 0.05).

Table 1 shows the average scores of the study and the control groups given by the subjects and the assessors in both interviews. As seen in table 1, the scores awarded by the assessors increased in the second interview for both the control and the study groups. In the study group, when total score (TS) was taken into account, the differences of *p*-value and CI values between the first and the second interview were significant. While examining the subheadings of the assessment in the study group, the mean values of total history scores, the history of present illness scores and the history taking ability scores differed significantly between the first and the second interviews. Other history components only show a small difference between the first and the second interviews at a level of α = 0.10 in the study group. On the other hand, for the control group, although the *p*-value of TS differed at the level of α = 0.10 cutting of CI to 1 decreased the importance of this difference. In the control group no significant difference was found between the first and the second interviews except for the mean history taking ability score (p = 0.002).

**Table 1:** The average scores of the study and the control groups given by the assessors and the students in both interviews.

Scores In Average		Assessors			Students		
		**1. time** **(Mean ± SD)**	**2. time** **(Mean ± SD)**	***p*-values**	**1. time** **(Mean ± SD)**	**2. time** **(Mean ± SD)**	***p*-values**

Total score (TS)	Study group	26.25 ± 6.71	31.57 ± 6.83	0.005	23.55 ± 7.85	26.61 ± 6.59	0.080
	
	Control Group	25.44 ± 6.40	29.13 ± 8.08	0.051	24.80 ± 7.73	29.86 ± 6.53	0.001

Total history score (THS)	Study group	15.29 ± 3.86	17.19 ± 3.60	0.021	14.18 ± 5.09	14.76 ± 3.79	0.569
	
	Control Group	15.32 ± 3.31	16.27 ± 4.44	0.380	14.84 ± 4.74	16.72 ± 4.64	0.094

History of present illness score (HPIS)	Study group	5.33 ± 1.70	6.52 ± 1.69	0.010	4.88 ± 2.08	5.42 ± 1.77	0.243
	
	Control Group	4.96 ± 1.74	6.00 ± 2.24	0.062	5.24 ± 2.00	6.04 ± 1.76	0.060

History taking ability score (HTAS)	Study group	5.07 ± 2.61	7.85 ± 2.72	0.000	4.48 ± 2.04	6.52 ± 2.33	0.001
	
	Control Group	4.56 ± 3.02	6.81 ± 3.06	0.002	5.28 ± 2.82	7.31 ± 2.60	0.000

Communication score (CS)	Study group	5.88 ± 1.55	6.52 ± 1.56	0.120	4.88 ± 1.80	5.33 ± 1.73	0.293

	Control Group	5.56 ± 1.15	6.04 ± 1.39	0.186	4.68 ± 1.90	5.81 ± 1.56	0.000

Other history components score (OHCS)	Study group	6.16 ± 2.56	7.19 ± 1.96	0.053	6.07 ± 2.51	5.85 ± 2.20	0.681
	
	Control Group	6.48 ± 2.14	6.95 ± 1.46	0.379	6.16 ± 2.56	7.59 ± 2.13	0.016


The scores awarded by the students also increased in the second interview for both the control and the study groups. Students' self- assessments show an almost opposite result from those given by their assessors. Differences of mean values of total scores, history scores, communication scores and other history components scores were significant for the control group, but were not significant for the study group.

There were no significant differences between the scores given by assessors for control and study groups of the two interviews. According to the students assessments in the first time there was no significant item but in the second time only for OHPC there was a significant difference between the study and control groups (p = 0.023).

## Discussion

Due to the assessments of the assessors the group having feedback both verbal and videotape were more successful than having verbal feedback alone. This concurs with other studies that have show that using videotaped consultation training by way of videotaping real consultations with subsequent feedback [10-12]. In the literature, there are many studies which indicate that videotaping is superior to other feedback methods [13-15].

Scores awarded by the assessors increased in the second interview for both the control and the study groups. This might be expected as a result of the feedback given to both groups in the first interview.

The absence of any difference between the two groups in their communication skills could be interpreted as a result of the educational program of our faculty. As a matter of standard, in DEUFM first year, students study communication skills via small group practices and role play throughout the first year.

Students' self- assessments show an almost opposite result from those given by their assessors. Are assessors giving better feedback based on their observations alone? This is not very likely. First of all, the study group also received feedback from the assessors just as the control group did, and additionally they also watched the video tapes of their interviews. So, if we were to accept that the students' assessments are more accurate than the assessors' assessments, then, we have to accept that video recording is a regressing method. This directly contradicts the literature. From the other side, the control group may be more perceptive giving feedback based on verbal feedback alone. Unlike the control group students, students in the study group had a chance to observe their own performances objectively. During the feedback sessions with the assessors, they could observe and focus on which aspects of their performance needed improvement, as well as in what areas they showed competency and excelled. This may have increased their self expectations. As a matter of fact, studies have shown that when students can watch video records of their performances with assessors, they become more aware of their deficiencies. Also in that study, students commented saying that they realized their "'lack of order" and "tendency to drift horizontally from one idea to the next", and the importance of constructing questions in "a directed fashion, rather than wandering aimlessly through a regimented history" [16].

The video-recording provided an excellent opportunity for students to observe his/her own performance, and its benefit was further enhanced by the comments of the assessor [17]. Although it plays a significant role in education, videotaping may also cause some problems. One of these shortcomings is the distress felt by students. Some studies show that students feel anxious and resist video recordings. On the other hand, these studies also show that this disadvantage could be overcome by initiating video based practices in the first year of medical education by watching previous video records of students, before they actually begin starting making their own, improving and positive feedback after video records and videotaping students in places that are informal and familiar to them [10,12,18,19]. There are also studies in which state that students wanted to perform more than one video record [20].

One of the limitations of the study was the small number of participants. The reason of that was a short time period between two interviews due to the ongoing education program of the students.

Second year students had received training in history taking and communication skills in the previous year. Although this is one of the limitations of the study, it is compensated by the fact that all students enrolled had received the same education.

Another limitation was that assessors were not blind to the subjects. This can be a threat to the validity of the findings, although it was tried to be minimized by the existence of well defined and strict rating criteria to reduce possible bias.

Videotaping feedback required more time than verbal feedback alone, so this may be another limitation of the study due to spending more time with assessor for the review and this may account for the difference between the groups.

## Conclusion

This study suggests that feedback based on videotaped interviews of students followed by students reviewing the tape with assessors afterwards, is superior to the feedback given solely based on the observations of assessors. However, study numbers limit the conclusions that can be drawn from the data.

## Competing interests

The authors declare that they have no competing interests.

## Authors' contributions

NO and DG conceived of the study and participated in its design, carried out and coordinate the study and drafted the manuscript. VM participated in the design of the study and performed the statistical analysis. TG, DY, ZS took place in study process and coordination. DG and IS edited the manuscript. All authors read and approved the final manuscript.

## Pre-publication history

The pre-publication history for this paper can be accessed here:

http://www.biomedcentral.com/1471-2458/9/474/prepub

## Supplementary Material

Additional file 1 **Assessment form**. Assessment form for the interviews of the students with the patients Click here for file
