# Supplementary material for: Is the use of videotape recording superior to verbal feedback alone in the teaching of clinical skills?
Source: BMC Public Health. 2009 Dec 19;9:474. doi: 10.1186/1471-2458-9-474 (PMC2808318; doi:10.1186/1471-2458-9-474)
Supplement: Additional file 1 — Assessment form. Assessment form for the interviews of the students with the patients [file 1471-2458-9-474-S1.DOC]

Assessment form for the interviews of the students with the patients

|  | |  | Insufficient  (0) | Sufficient  (1) | Successful  (2) |
| --- | --- | --- | --- | --- | --- |
| Communication Skills | | 1. Getting acquainted with the patient. (Does he/she introduce himself/herself to him/her? Shaking hands? Does he/she ask his/her name? |  |  |  |
|  |  | 1. Does he/she set up a communication level by making the patient feel better? |  |  |  |
|  |  | 1. Does he/she utilize the communication skills? (reflection / empathy / respect / support) |  |  |  |
|  |  | 1. Facilitating skills. (Does he/she make eye contacts? Is his/her speech clear?) |  |  |  |
| History taking ability | | 1. Does he/she start with open-ended questions and end with closed-ended questions? |  |  |  |
|  |  | 1. Does he/she set up a history-composition? |  |  |  |
|  |  | 1. Does he/she make chapter summaries by orienting the patient towards his/her goal? |  |  |  |
|  |  | 1. Does he/she transfer the explanatory information to the patient in a simple way and language? |  |  |  |
|  |  | 1. Does he/she make summaries and clarification |  |  |  |
|  |  | 10. Does he/she make regulations for next step and end visit properly? |  |  |  |
| Total history |  | 11. Identification data |  |  |  |
|  |  | 12. Main complaint |  |  |  |
|  | History of present illness | 13. Primary history |  |  |  |
|  |  | 14. Secondary history |  |  |  |
|  |  | 15. Tertiary history |  |  |  |
|  |  | 16. Patient perspective |  |  |  |
|  |  | 17. Things have done for the present illness |  |  |  |
|  | Other history components | 18. Past History |  |  |  |
|  |  | 19. Current Health Status |  |  |  |
|  |  | 20. Family History |  |  |  |
|  |  | 21. Personal and Social History |  |  |  |
|  |  | 22. Review of Systems |  |  |  |
